# Hypoxia-inducible factor-1alpha is a critical mediator of hypoxia induced apoptosis in cardiac H9c2 and kidney epithelial HK-2 cells

**DOI:** 10.1186/1471-2261-8-9

**Published:** 2008-04-30

**Authors:** Ricky Malhotra, David W Tyson, Henry M Rosevear, Frank C Brosius

**Affiliations:** 1 Department of Internal Medicine/Nephrology, University of Michigan Medical Center, Ann Arbor, MI-48109-0676, USA; 2 Department of Molecular and Integrative Physiology University of Michigan Medical Center, Ann Arbor, MI-48109-0676, USA

## Abstract

**Background:**

Hypoxia inducible factor-1 (HIF-1) is a transcription factor that functions to maintain cellular homeostasis in response to hypoxia. There is evidence that HIF-1 can also trigger apoptosis, possibly when cellular responses are inadequate to meet energy demands under hypoxic conditions.

**Methods:**

Cardiac derived H9c2 and renal tubular epithelial HK-2 cells expressing either the wild type oxygen regulated subunit of HIF-1 (pcDNA3-Hif-1α) or a dominant negative version that lacked both DNA binding and transactivation domains (pcDNA3-DN-Hif-1α), were maintained in culture and exposed to hypoxia. An RNA interference approach was also employed to selectively knockdown expression of Hif-1α. Apoptosis was analyzed in both H9c2 and HK-2 cells by Hoechst and TUNEL staining, caspase 3 activity assays and activation of pro-apoptotic Bcl2 family member Bax.

**Results:**

Overexpression of pcDNA3-DN-Hif-1α led to a significant reduction in hypoxia -induced apoptosis (17 ± 2%, *P *< 0.01) in H9c2 cells compared to both control-transfected and wild type Hif-1α transfected cells. Moreover, selective ablation of HIF-1α protein expression by RNA interference in H9c2 cells led to 55% reduction of caspase 3 activity and 46% reduction in the number of apoptotic cells as determined by Hoechst 33258 staining, after hypoxia. Finally, upregulation of the pro-apoptotic protein, Bax, was found in H9c2 cells overexpressing full-length pcDNA3-HA-HIF-1α exposed to hypoxia. In HK-2 cells overexpression of wild-type Hif-1α led to a two-fold increase in Hif-1α levels during hypoxia. This resulted in a 3.4-fold increase in apoptotic cells and a concomitant increase in caspase 3 activity during hypoxia when compared to vector transfected control cells. HIF-1α also induced upregulation of Bax in HK-2 cells. In addition, introduction of dominant negative Hif-1α constructs in both H9c2 and HK-2 -cells led to decreased active Bax expression.

**Conclusion:**

These data demonstrate that HIF-1α is an important component of the apoptotic signaling machinery in the two cell types.

## Background

Hypoxia-inducible factor -1 is a transcription complex composed of an alpha (Hif-1α) and a beta (Hif-1β) subunit [1]. While Hif-1β is constitutively expressed, Hif-1α levels are highly regulated by cellular oxygen tension and determine the cellular responses to variations in the oxygen tension [2,3]. Whenever Hif-1α is induced in a cell in response to low oxygen conditions it primarily triggers a number of pro-survival events such as increased expression of vascular endothelial growth factor (VEGF) and hence angiogenesis, increased expression of genes for various glycolytic enzymes as well as for the glucose transporter 1 (GLUT1) thereby enhancing glycolysis, and activation of the erythropoietin gene and hence erythropoiesis [4].

Over the past decade it has become clear that hypoxia induces programmed cell death, or apoptosis, through a mitochondrial dependent process [5,6] involving release of cytochrome c, activation of caspase 9 and subsequent cleavage and activation of downstream, effector caspases [7,8]. Multiple factors are involved in this programmed cell death pathway but because it largely determines the cellular response to hypoxia, Hif-1 has been a candidate to also regulate hypoxia-induced apoptosis. Despite its clear role in cellular protection, there have been a number of reports demonstrating that Hif-1 also contributes to programmed cell death during hypoxia [e.g., [9-13]]. Recent reports have centered on the effects of Hif-1 on transactivation of the BNIP3 gene in certain cells such as cardiac myocytes [11,13,14]. The BNIP3 gene product induces apoptosis by binding and inhibiting the antiapoptotic proteins Bcl-2 and Bcl-xL [11,13,14]. However, BNIP3 is not present in all cells, and Hif-1 clearly does not induce apoptosis in many cell types. For example, Hif-1 prevents hypoxia-induced apoptosis in pancreatic cancer cells [15-19]. Thus, the role of Hif-1 in apoptosis remains somewhat controversial and appears to be cell type and context specific.

Previously, we have found that enhanced glycolytic metabolism and GLUT1 expression markedly attenuate hypoxia induced apoptosis [20-22] and that enhanced glycolytic metabolism and GLUT1 expression suppress levels of Hif-1α in hypoxic cardiac myocytes [23]. Recently hyperglycemia has been demonstrated to inhibit HIF-1α expression, inhibit hypoxia-induced decreased proliferation of vascular smooth muscle cells and a concomitant decrease in hypoxia response element (HRE) promoter transactivation [24]. We therefore hypothesized that reduction of Hif-1α induced by GLUT1 and glucose metabolism was mechanistically important in the prevention of apoptosis and that Hif-1 plays a proapoptotic role in our systems, especially in the absence of glucose transport. To test this latter hypothesis we determined the effect of Hif-1α expression in both a cardiac myocyte cell line and a kidney epithelial cell line. The rationale for using two different cell lines was to underscore the importance of Hif-1 in two distinct cell types which are clinically relevant for apoptosis in cardiac (H9c2) and renal (HK-2) ischemia. We found that Hif-1 enhances apoptosis and that hypoxia-induced apoptosis can be markedly suppressed by either a dominant negative Hif-1α construct or shRNA induced knockdown of Hif-1α. Hif-1α expression is also associated with enhanced Bax activation in these systems thereby providing a mechanistic link between hypoxic stress and apoptosis in these two cell types.

## Methods

### Cell culture

The embryonic rat heart derived H9c2 cells were cultured in DMEM (Gibco) supplemented with 10% fetal bovine serum (Gibco), antibiotics (50 U/ml penicillin and 50 μg/ml streptomycin) at 37°C in an atmosphere of 95% air and 5% CO_2_. The human kidney epithelial cell line HK-2 (ATCC #CRL-2190) was grown in keratinocyte serum-free basal medium (Gibco) supplemented with bovine pituitary extract (50 μg/ml), recombinant epidermal growth factor (5 μg/ml), 50 U/ml of penicillin and 50 μg/ml streptomycin.

### Establishment of hypoxic culture condition

Both H9c2 and HK-2 cells were grown to the desired confluence in a regular tissue culture incubator and then transferred to an air tight Plexiglas hypoxic chamber in glucose-free medium containing 2% Oxyrase (0.025% for HK-2 cells) as described previously [20]. Establishment of environmental hypoxic conditions (< 1%) were achieved by continuously flushing the chamber with a water saturated mixture of 5% CO_2 _and 95% N_2_. Maintenance of the desired O_2 _concentration was constantly monitored during incubation using a microprocessor-based oxygen sensor.

### Generation of Constructs

The original Hif-1α construct containing the full length human Hif-1α cDNA, nt 1–3389 (pCEP4/Hif-1α T7) was obtained from Dr. Greg Semenza (Johns Hopkins, Baltimore, Maryland). The mammalian expression constructs encoding the HA-tagged version of wild type Hif-1α (pcDNA3-HA-Hif-1α) and the Flag-tagged form of human dominant negative (DN) Hif-1α (pcDNA3-Flag-DN-Hif-1α) were generated by sub-cloning, respectively, the full length human Hif-1α cDNA or a truncated Hif-1α cDNA, in which the sequence encoding the N-terminal DNA binding domain (amino acids #1–17) and the entire C-terminal transactivation domain (amino acids #401–826) was deleted by PCR, into the EcoR1 and XbaI sites of the pcDNA 3 mammalian expression vector tag series containing the HA or Flag epitope subcloned between the HindIII and the EcoRI site, as previously described [25].

### Transfection and Immunoblot analysis

H9c2 cells and HK-2 cells were seeded in 60 mm plates. When the cells reached 50–60% confluence, transfection with 500 ng-1 μg of control vector (pEGFP) or one of the wild type or dominant negative Hif-1α constructs was carried out using Fugene (Roche Biochemicals) according to the manufacturer's protocol. Transfection efficiencies were monitored by counting EGFP positive cells. In H9c2 and HK-2 cells the average transfection efficiency of six independent experiments (n = 6) was 22 ± 5% and 48 ± 7.6% respectively. Two days after transfection, H9c2 cells were subjected to 6–24 h of hypoxia in regular DMEM, deficient in glucose, but supplemented with 10% FCS. For HK-2 cells, hypoxia of 16 h was performed in complete keratinocyte basal serum-free medium as outlined above.

Whole cell lysates were prepared according to standard protocol. Cells were extracted with lysis buffer (50 mmol/L Tris-HCl, pH 7.5, 5 mmol/L EDTA, 150 mmol/L NaCl, 0.5% Triton X-100, 10 mmol/l NaF, 0.1% SDS, 250 μmol/L sodium orthovandate, 1 mmol/L PMSF and Complete Protease Inhibitor Cocktail [Roche Biochemicals]), and incubated at 4°C for 30 min. The lysates were sonicated and centrifuged at 14,000 *g *for 30 min. The supernatants were collected and stored at -70°C or used immediately. Protein concentrations were determined by the BCA (Pierce Biochemicals) method. Protein (100 μg) was separated on 6% (for Hif-1α detection) or 10% (for Bax detection) polyacrylamide-SDS gel and blotted onto nitrocellulose membranes (Hybond ECL, Amersham, Pharmacia). After blocking with TBS/5% skim milk, the membranes were incubated overnight at 4°C with primary antibodies against Hif-1α (1:500 dilution, Novus Biologicals, Littleton, CO), the active monomer of Bax (Monoclonal antibody 6A7, 0.5 μg/ml concentration, Alexis Biochemicals, San Diego, CA) or total Bax (1:1000 dilution, Santa Cruz Biotechnology, Santa Cruz, CA), followed by secondary antibodies conjugated with horseradish peroxidase (1:10,000) (Pierce, Rockford, IL) for 1 h at room temperature. Signals were detected with ECL.

### shRNA treatment of cells

Multiple short hairpin (sh) RNA oligonucleotides to human HIF-1α sequence were synthesized using an algorithm for rational siRNA design [26]. The top and bottom cDNA oligomers with a 4 nt loop sequence were annealed to generate a double stranded oligo with 4-nucleotide overhangs which was then used for directional cloning into the BLOCK-iT™ U6 RNAi Entry vector kit (Invitrogen Life Technologies). After completion of cloning the U6 entry vector with the inserted RNAi cassette was transiently transfected into cells using Fugene (Roche Biochemicals). shRNA oligos which generated optimal silencing of Hif-1α had the following complementary strand sequences : 5'-AGAGGUGGAUAUGUGUGGGdTdT-3' and 5'-CCAACACAUAUCCACCUCUdTdT-3'.

### Apoptosis analysis

Assessment of cellular apoptotic morphologies after transfection and hypoxia was accomplished by plating the H9c2 and HK-2 cells in 2 well Falcon glass culture slides (Becton Dickinson Labware, Franklin Lakes, NJ). pEGFP was co-transfected with the other constructs to aid in the identification of transfected cells for counting purposes. The cells were rinsed in PBS, pH 7.4, and fixed for 30 min in 4% paraformaldehyde in PBS at room temperature, and rinsed again in PBS. The cells were then permeabilized in 0.1% Triton X-100 in 0.1% sodium citrate. The cells were rinsed in PBS and then stained directly with anti-HA-fluorescein or anti-Flag-fluorescein monoclonal antibodies (Roche Biochemicals). After rinsing in PBS the cells were also stained with the karyophillic dye Hoechst 33258 (5 μg/ml) for 10 min at room temperature. After a final rinse in PBS the cells were mounted in moiwol (Polysciences, Inc., Warrington, PA), an antifade agent, and visualized under fluorescent light (for detection of EGFP, HA and Flag epitopes respectively) and switched to ultraviolet light (for visualization of Hoechst 33258) with a Leitz Orthoplan microscope. The percentage of EFGP/HA/Flag positive cells displaying chromatin condensation and nuclear fragmentation was determined.

### TUNEL staining

Further characterization of apoptosis in HK-2 cells was performed using a commercially available *in situ *cell death detection kit to find DNA strand breaks using the terminal deoxynucleotidyl transferase-mediated dUTP nick end labeling (TUNEL) reagent according to the manufacturer's protocol (Boehringer Mannheim). The number of TUNEL-positive cells was counted in five different fields by an observer blinded to cell treatment or transfection status.

### Caspase 3 activity assay

Caspase 3 activity was assessed via a colorimetric assay utilizing specific substrates (Calbiochem). After treatment, the cells were washed once with ice-cold PBS and collected by trypsinization followed by centrifugation. The cellular pellet was resuspended in cell lysis buffer and incubated on ice for 10 min. Lysates were centrifuged for 5 min at 13,000 rpm, and the supernatants were assayed for caspase 3 activity in assay buffer (50 mM HEPES, pH 7.4, 100 mM NaCl, 0.1% CHAPS, 10 mM dithiothreitol, 0.1 mM EDTA, 10% glycerol). After addition of DEVD-specific caspase substrate (2 mM) samples were incubated for 60 min at 37°C and read at 405 nm in an EL-312 Bio-Kinetics microplate reader (Bio-Tek Instruments).

### Flow cytometry analysis and sorting

H9c2 and HK-2 cells were transfected with both pEGFP and pcDNA3-HA-Hif-1α as described above. After 24 h of transfection the cells were trypsinized and suspended in Dulbecco's PBS at a concentration of at least 1 × 10^6 ^cells/ml. The cells were analyzed and sorted in a Flow cytometer (FACS Vantage SE, Becton Dickinson) to obtain only EGFP positive cells based on laser excitation wavelengths of 488 nm and emission wavelength of 525 nm. The sorted cells were allowed to acclimatize for a few hours in a tissue culture incubator and then subjected to hypoxia of 6 h, followed by immunoblotting to examine active Bax as well as total Bax in total cellular lysates as described above.

### Statistics

Data were expressed as mean ± S.E. unless otherwise indicated in the figure legends. Analysis of variance was followed by Bonferroni's post-hoc analysis, and differences were regarded as significant at a *p *value < 0.05.

## Results

### Overexpression of a dominant negative Hif1-1α reduces hypoxia-induced apoptosis in H9c2 cells

**Figure 1:**
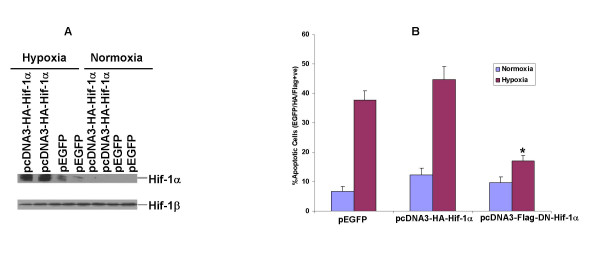
A) Hif-1 α protein expression in whole cell lysates of H9c2 cells transfected with either pEGFP or pcDNA3-HA-HIF-1α and subjected to normoxia or hypoxia for 12 h. Immunoblotting with antibodies against HIF-1β, the nuclear dimerization partner of HIF-1α was used to monitor equal protein loading. B) HIF-1α mediates hypoxia-induced apoptosis in H9c2 cells transfected with pEGFP or pcDNA3-HA-HIF-1α expressing the wild type HIF-1α or pcDNA3-Flag-DN-HIF-1α expressing the dominant negative version as described in methods. Cells were exposed to normoxia or hypoxia for 12 h in glucose-deficient medium. The number of cells displaying condensed and fragmented nuclei was counted only in cells positive for each respective epitope tag or EGFP. The results are representative of five independent experiments. *P < 0.01 vs. hypoxia in pEGFP and pcDNA3-HA-Hif-1α.

H9c2 cells were transfected with either control vectors or wild-type or dominant negative Hif-1α constructs. Under normoxic conditions there was no increase in the number of apoptotic cells or detectable Hif-1α levels after transfection with either the wild type or truncated Hif-1α constructs. These findings were consistent with proteasomal degradation of Hif-1α in the presence of O_2_, as the oxygen degradation domain is intact in both the wild type and dominant negative constructs. (Fig. 1A). However overexpression of the dominant negative Hif-1α that lacks both DNA binding and transactivation domains significantly reduced the number of apoptotic cells during hypoxia (17+2% in dominant negative cells, 44.7+4.4% apoptotic cells in wild type Hif-1α transfected cells, and 38.5+3.8% in EGFP transfected cells) (Fig. 1B). Thus, in H9c2 cells Hif-1 seems to be partly responsible for the observed hypoxia-induced apoptosis in the absence of glucose.

### Knock down of hypoxia-induced expression of Hif-1α with RNA interference (RNAi) reduces hypoxia induced apoptosis in H9c2 cells

**Figure 2:**
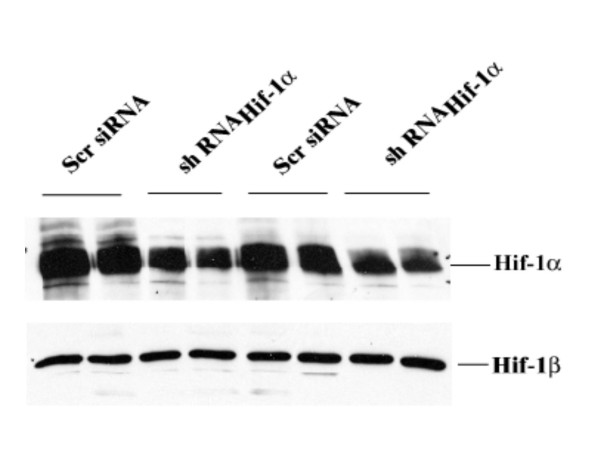
Effect of shRNA treatment on HIF-1α protein expression in H9c2 cells. H9c2 cells were transfected with shRNAi_HIF-1α _(200 nM) or an shRNAi targeted to a scrambled mRNA sequence (Scr siRNA, nonspecific control), and 24 h later were subjected to hypoxia for an additional 24 h and subsequently whole cell lysates were isolated for Western blot analysis for HIF-1α protein expression. The data shown is from two independent experiments. As a loading control the lysates were also probed with an antibody against Hif-1β, the dimerization partner of Hif-1α.

**Figure 3:**
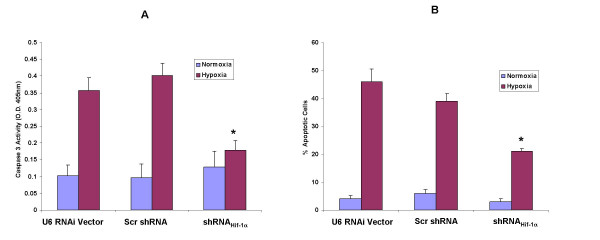
Downregulation of HIF-1α by shRNAi treatment reduces hypoxia-induced apoptosis in H9c2 cells. After co-transfection with shRNA_HIF-1α _or an shRNAi targeted to an unrelated mRNA sequence (Scr shRNA, nonspecific control) or with the U6RNAi empty vector and pEGFP the H9c2 cells were cultured under normoxic or hypoxic conditions for an additional 24 h. (A) After transfection with respective shRNAi constructs and exposure to normoxic or hypoxic conditions the cells were analysed for caspase 3 activity. Results are mean ± SEM of 4 separate experiments. (B) Cells were stained with Hoechst 33258 and visualized under ultraviolet light. The data represent the mean ± SEM of triplicate wells of three independent experiments. *P < 0.05 vs. hypoxia in U6 RNAi vector and Scr shRNAi.

To confirm the specificity of the cellular apoptosis response to Hif-1 seen in H9c2 cells, an alternative approach using shRNAi_HIF-1α _was employed. When transfected into cells, siRNA-Hif-1α targets HIF-1α mRNA for degradation, thus blocking HIF-1 activity [26]. H9c2 cells were treated with shRNAi_Hif-1α _for 24 h followed by 24 h of hypoxia and finally immunoblotting to assess Hif-1α protein expression. Under hypoxic conditions Hif-1α protein was suppressed in H9c2 cells treated with shRNAi_Hif-1α _(200 nM) when compared to cells transfected with shRNA targeted to a scrambled mRNA sequence (negative control) (Fig. 2). Densitometry analysis revealed a 76 ± 8.5% decrease in Hif-1α levels in cells transfected with shRNAi_Hif-1α _compared to cells transfected with shRNA targeted to a scrambled sequence (*P *< 0.001, n = 3). The selective reduction of Hif-1α levels resulted in a decrease in apoptosis as documented by a reduction of caspase 3 activity by 55% compared to negative controls and cells transfected with the empty U6RNAi vector alone (*P *< 0.01) (Fig 3A). Similarly, the number of cells that were morphologically apoptotic, based on staining with the karyophillic dye Hoechst 33258, were decreased 46% by shRNAi_Hif-1α _treatment (p < 0.05). (Fig. 3B). These findings confirmed that a decrease in apoptosis was a specific result of reduction of active Hif-1α.

### Hif-1α promotes Bax activation in H9c2 cells

**Figure 4:**
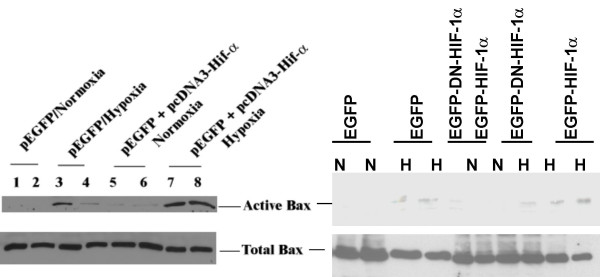
Flow cytometry analysis followed by Bax protein expression. H9c2 cells were transfected with pEGFP, pEGFP and pcDNA3-HA- HIF-1α or pcDNA3-Flag-DN-HIF-1α and were sorted in a flow cytometer. The EGFP positive sorted cells were subjected to hypoxia for 6 h. Total Bax expression in the same lysates was used to monitor protein loading. N = Normoxia; H = Hypoxia. The data are representative of 3 separate experiments and only 2 experiments using dominant negative Hif-1α (right panel).

To gain insight into the mechanisms underlying apoptosis by Hif-1α in H9c2 cells, the induction of the proapoptotic protein Bax was investigated. The 6A7 antibody detects an epitope at the N terminus of Bax that is occluded in the inactive state and is exposed upon Bax activation [27]. H9c2 cells, transfected with pEGFP, DN and wild-type Hif-1α vectors, were selected as described in Materials and Methods. Whole cell lysates of H9c2 cells expressing EGFP and wild type Hif-1α demonstrated a strong activation of Bax, while lysates of cells expressing EGFP alone displayed little Bax activation (Fig. 4). Interestingly, H9c2 cells transfected with DN Hif-1α displayed reduced Bax expression thereby suggesting that Hif-1α was responsible for Bax activation. Although we did not observe any significant increase in apoptosis under similar transfection conditions (Fig. 1) these results suggest that Hif-1α may promote Bax activation during hypoxia. Under normoxic conditions active Bax was not detected in cells transfected with pEGFP alone or pEGFP plus Hif-1α (Fig. 4).

### Hif-1α overexpression induces apoptosis in human kidney epithelial HK-2 cells

**Figure 5:**
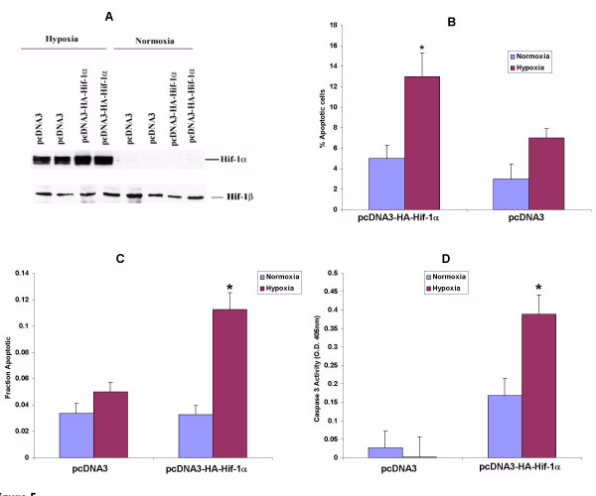
Effect of HIF-1α overexpression on apoptosis in HK-2 subjected to 16 h of hypoxia. (A) HIF-1α protein expression in HK-2 cells transfected with either the empty vector or pcDNA3-HA-Hif1α and subjected to hypoxia or normoxia. Immunoblotting of the same lysates with Hif-1β to monitor protein loading is also shown. (B) Morphological analysis of apoptosis with Hoechst 33258. *P < 0.05 vs hypoxia in pcDNA3. pEGFP was co-transfected alongwith the vector and HIF-1 alpha construct to aid in the identification of apoptotic nuclei in EGFP transfected cells. (C) Apoptotic cells as determined by TUNEL staining. *P < 0.001 vs hypoxia after transfection with pcDNA3. Data shown is mean ± SEM of six separate experiments and (D) Caspase 3 activity (O.D. units 405 nm).*P < 0.001 vs. hypoxia with the empty vector pcDNA3. Data in panels B, C and D are means ± SEM of triplicate wells of six separate experiments.

We also extended our studies on Hif-1α and apoptosis to the kidney epithelial cell line, HK-2. Transfection of HK-2 cells with a native Hif-1α construct resulted in an almost two fold increase in total Hif-1α levels in the cells transfected with wild-type Hif-1α (Fig. 5A). Using a combination of three approaches to document apoptosis in these cells, we found that transfection with wild type Hif-1α followed by hypoxia of 16 h resulted in substantial apoptosis whereas, under these conditions at least, there was no significant increase in apoptosis in hypoxic vector-control transfected cells (Fig. 5 B–D). These findings are somewhat different than those in H9c2 cardiac cells which demonstrated substantial hypoxia-induced apoptosis that was not augmented by Hif-1α overexpression, but which was ameliorated when Hif-1α was reduced or inhibited.

### Increased total Hif-1α and active Bax in Hif-1α transfected HK-2 cells

**Figure 6:**
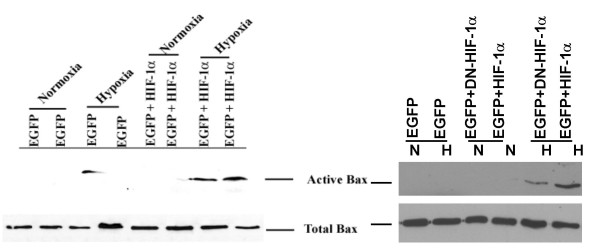
HIF-1α promotes Bax activation in HK-2 cells. Cells were subjected to normoxia or hypoxia of 16 h after sorting in a flow cytometer. Active and Total Bax protein expression of the whole cell lysates of EGFP positive cells is shown of cells transfected with either pEGFP or pEGFP and pcDNA3-HA-Hif-1α or pcDNA3-DN-Hif-1α. N = Normoxia; H = Hypoxia. Data shown is representative of two separate experiments.

We further explored whether Hif-1α overexpression was also associated with increased Bax activation in HK-2 cells. HK-2 cells were transfected with vector alone (pEGFP) with wild type Hif-1α and pEGFP or with DN Hif-1 and EGFP as described above. EGFP positive cells were allowed to acclimatize in the tissue culture incubator and subsequently subjected to 16 h hypoxia or normoxia. Immunoblotting of the same lysates demonstrated an upregulation of active Bax in cells transfected with pcDNA3-HA-Hif-1α compared to cells transfected with DN-Hif-α (Fig. 6), suggesting that Hif-1 enhanced Bax activation during hypoxia and DN Hif-1α was effective in reducing both Bax expression and the resultant apoptosis.

## Discussion

Hif-1 is critical for the cellular response to hypoxia as it serves to transactivate a number of genes responsible for cellular survival [4]. Conversely, Hif-1 can also stimulate the mitochondrial apoptotic pathway and cell death during hypoxia, [e.g., [9-11,13]]. However, the role of Hif-1 in hypoxia-induced apoptosis remains controversial, and several reports have found that Hif-1 actually protects some cell types from apoptosis, presumably in a cell-type and context specific manner [e.g., [15-19]]. We previously found that glucose uptake and metabolism or enhanced expression of the facilitative glucose transporter, GLUT1, protected cardiac myocytes and other cell types from hypoxia-induced apoptosis via reduced mitochondrial release of cytochrome c and activation of caspase 9 [20-22]. In addition, we found that enhanced glucose uptake and metabolism or increased GLUT1 expression resulted in reduced Hif-1α levels due to enhanced Hif-1 α proteosomal degradation despite persistent hypoxia in these same cardiac myocytes and heart-derived H9c2 cells [23]. A similar effect of hyperglycemia on reduction of Hif-1α levels and apoptosis has been demonstrated in vascular smooth muscle cells [24]. In a rat model of cerebral ischemia both mRNA and protein expression of Hif-1α and procaspase 3 was also shown to be increased after 12- and 24-hours of ischemic insult and increase in specific Hif-1 binding to the caspase 3 gene promoter [28]. Therefore, we hypothesized that Hif-1 promoted mitochondrial cell death pathway in H9c2 cells and that glucose-induced reduction in Hif-1α levels contributed to the anti-apoptotic effects of glucose uptake and metabolism.

The present report demonstrates that Hif-1α potentiates apoptosis under hypoxic conditions in both rat H9c2 cells and human renal epithelial HK-2 cells. Overexpression of a dominant negative Hif-1α that lacked DNA binding domain and the C-terminal transactivation domain as well as shRNA targeted to Hif-1α decreased hypoxia-induced apoptosis in H9c2 cells. Conversely, overexpression of wild type Hif-1α increased apoptosis in HK-2 cells, whereas it had little effect in H9c2 cells, suggesting that levels of Hif-1α in these cells during hypoxia were already sufficient to maximally trigger apoptosis. These differences may reflect the relative levels of Hif-1α in the two cell lines and also the threshold of Hif-1α overexpression needed to trigger a significant apoptotic response. Thus, in HK-2 cells with the existing endogenous levels of Hif-1α, hypoxia could have resulted in relatively small increases in caspase activity and apoptosis, but augmenting Hif-1 α levels boosted those apoptotic features. On the other hand, in H9c2 cells overexpression of Hif-1α had little effects on apoptosis, but reduction of Hif-1α inhibited apoptosis.

These observations are consistent with other studies showing that Hif-1 promotes apoptosis in cardiac myocytes and other cells by induction of the cell death factors BNIP3 and NIX [11,13,29,30] or by stabilizing the tumor suppressor protein p53 [31]. As noted above, however, several studies have reported that Hif-1α is a prosurvival factor and can protect cells from hypoxia-induced apoptosis [15,17-19]. Most of these latter studies have been performed in tumor or tumor derived cells in which adaptation to the hypoxic microenvironment leads to the selection of certain malignant tumor phenotypes, which may select Hif-1 as a survival factor.

In cardiac myocytes and kidney epithelial cells which under normal circumstances are not normally exposed to a hypoxic microenvironment, Hif-1 may serve dual roles as a survival and death factor depending upon the severity and duration of insult or injury. For example, a recent study in which primary cardiac myocyte cultures were exposed to metabolic inhibition with cyanide and 2-deoxyglucose documented survival benefits of inhibiting the family of the O_2_-dependent prolyl hydroxylase (PHD) enzymes responsible for targeting Hif-1α for proteosomal degradation [32]. These inhibitors stabilize Hif-1α levels. This report suggested that under certain circumstances, Hif-1 can be anti-apoptotic in cardiac myocytes. However, there may have been some nonspecific effects in this study since the two inhibitors used in this report were used at concentrations somewhat higher than in most other reports (500 μM) and would have affected levels of other proteins. Nonetheless, other recent studies have corroborated the protective effects of HIF-1-dependent pathways in cardiac myocytes by overexpressing constitutively stable hybrid forms of HIF-1α or with small interfering RNA (siRNA) directed against HIF-1α-prolyl-4–hydroxylase-2 gene, thus stabilizing HIF-1α [33,34]. Why our results differ from these previous reports is unclear, and could be due to the use of somewhat different cell systems and conditions. One important difference is that our systems combined both true hypoxia with glucose withdrawal. Since we and others have clearly shown that glucose uptake and metabolism helps protect cells from various extracellular stresses including hypoxia and growth factor withdrawal-induced apoptosis [20-22,35-37], the absence of this protective response will allow greater Hif-1α activity [23] and possibly potentiate its pro-apoptotic activity.

Hypoxia has also been shown to activate the Unfolded Protein Response (UPR), an essentially adaptive response that occurs in the endoplasmic reticulum (ER) in response to stresses that reduce or alter protein folding [38,39]. Though recent reports demonstrate that hypoxia activates the UPR in cardiac myocytes [40] which may potentially protect the myocardium during hypoxic stress, the fact that in the event of an unresolved ER stress the UPR can also activate the apoptotic cell death program [41,42] also needs to be addressed.

Finally, our studies suggest, but do not prove, that Hif-1α promotes apoptosis by activating the proapoptotic Bcl-2 family protein Bax. Previous reports have suggested that Hif-1 is necessary for hypoxia-induced Bax expression [43]. However, other reports have not substantiated this relationship [16]. Consistent with our observations, a recent report also demonstrates hypoxia-mediated apoptosis in temperature sensitive rat proximal tubular cells through a mechanism independent of Bax upregulation at least at the permissive temperature [44]. Whether Hif-1 directly activates Bax gene expression or induces Bax expression through some intermediate factor or factors is not clear and needs further investigation. In both H9c2 and HK-2 cells Hif-1 expression leads to upregulation of active Bax and introduction of dominant negative constructs of Hif-1α leads to reduced Bax expression. Whether Bax directly contributed to increased apoptosis in HK-2 and H9c2 cells still needs to be addressed. One obvious candidate which could be involved in Bax induction is the tumor suppressor protein p53. Numerous studies have documented that Hif-1 stabilizes p53 [31,45] and that p53 can induce Bax translocation and thus engage the mitochondrial cell death pathway [46,47]. Simultaneously hypoxia leads to increased mitochondrial reactive oxygen species (ROS) generation [48] and previous reports have convincingly demonstrated that these mitochondrial ROS are both sufficient and necessary to activate HIF [49]. Activation of HIF-1a by a redox-senstive pathway has also been shown to promote mitochondrial cell death pathway by upregulating BNIP3 [50] thereby providing a complex regulatory network where HIF-1α induction either upstream or downstream of the mitochondria finally leads to increased expression of mitochondrial death factors (Bax, BNIP3 etc) and resultant apoptosis.

## Conclusion

Hif-1 plays a master regulatory role in the cellular response to hypoxia. In certain circumstances and in certain cell types, Hif-1 promotes apoptosis in the presence of hypoxia, especially when other cellular energy substrates are lacking.

## Competing interests

The authors declare that they have no competing interests.

## Authors' contributions

RM helped designed the study, carried out the cell and molecular experiments on cardiac H9c2 cells and wrote the manuscript. DJWT helped in engineering the dominant negative Hif-1α constructs and maintained the H9c2 cells in culture. HMR performed the experiments on HK-2 cells. FCB participated in the design and coordination of the study and also participated in the writing of the manuscript. All authors read and approved the final manuscript.

## Pre-publication history

The pre-publication history for this paper can be accessed here:


